# On the Pathology and Treatment of Dysentery, Being the Gulstonian Lectures, Delivered at the College of Physicians in February, 1847

**Published:** 1847-10

**Authors:** 


### Art. III.

1. *On the Pathology and Treatment of Dysentery, being the Gulstonian Lectures, delivered at the College of Physicians in February,* 1847. By William Baly, m.d., Physician to the Milbank Prison, and Lecturer on Forensic Medicine at St. Bartholomew's Hospital. From the Medical Gazette.—*London,* 1847. 8vo.
2. *Observations on the History and Treatment of Dysentery and its Combinations, &c. &c.* By William Harty, m.d., Physician to the King's Hospital and to the Prisons of Dublin. Second Edition.—*Dublin,* 1847. 8vo. pp. 298.
3. *Notes on the Pathology and Treatment of Dysentery, as observed in the European General Hospital at Bombay during the Five Years from July,* 1838, *to July,* 1843. By C. Morehead, m.d. (Transactions of the Medical and Physical Society of Bombay, No. VII. Presented April, 1845.)
4. *A Dictionary of Practical Medicine.* By James Copland, m.d., f.r.s. Vol. I. Art. Dysentery.—*London,* 1844. 8vo, pp. 1056.

Perhaps there is no single disease that has received a more voluminous illustration at the hands of writers than dysentery. And although the European practitioner of the present day has seldom occasion to witness the disease in that severe and epidemic form which called forth the observations of Sydenham, Willis, and Morton, or, at a later date, of Huxham, Zimmermann, Durandeau, or Degner, although that concourse of unfavorable hygienic conditions which in London, in the 17th century, caused dysentery to be one of the most fatal diseases in the bills of mortality,* no longer exists, still, even in temperate climates, the active causes of the diseases are but dormant, not eradicated, and are therefore not incapable of development. A famine in Ireland may recall to us, for a time, those scenes which the general misery of the population in London rendered familiar to Sydenham; and the military surgeon of our own day has witnessed in the track of the French armies, or even of our own troops in Spain and Egypt, the same devastations and the same mortality described by Pringle in the campaigns in Flanders. And although, except in such circumstances, dysentery has ceased to be a complaint daily witnessed at our own doors, this disease is still well known to the subjects of a maritime power like England, in whose vast colonies, situated in every climate and on every soil, it is certainly the most fertile in bad consequences, if not the most rapidly fatal and destructive. If cholera and yellow fever are unparalleled in the tremendous rapidity of their action, and in their resistance to the powers of medicine, dysentery is no less formidable from its oftentimes insidious nature, from its tendency to recur, and from the after-influences it exerts on particular organs, or on the system at large. Therefore almost all writers on the diseases prevalent in the colonial possessions of England have placed dysentery at the head of the list of severe affections, and have referred to it as the cause and

* The deaths from 1667 to 1692, recorded under the heads of "bloody flux" and "griping of the guts," amounted to 2000 per annum. (Heberden on the Increase and Decrease of different Diseases in London, 1801.)

origin of many of those chronic and intractable abdominal diseases witnessed so often in Europeans resident in tropical climates.

In their accounts of this disease, the best writers have differed among themselves. Pringle remarks of Sydenham and Willis, that in their description of dysentery they scarcely agree in anything beyond the name; and if the differences among more recent authors are less conspicuous, they are still numerous and important. This discordance apparently arises from the fact—that dysentery, although *per se* a simple and uniform disease, is yet liable to constant changes in type, from differences in the causes which produce it; in other words, from alliances with other diseases. To a disquisition on dysentery, considered in this point of view, the first edition of Dr. Harty's work, published in 1805, was devoted; and the expected prevalence of dysentery at the present time in Ireland has induced him to publish a second edition, in which, with some important alterations, his former views are ably argued and sustained. The following propositions present a summary view of the conclusions he has arrived at:

"1st. That the genuine and simple dysentery is unattended by idiopathic fever, and is never of itself contagious.

"2d. That every other form of the disease, when epidemic, is a combination of the simple dysentery either with intermittent, remittent, or continued fever.

"3d. That the combination with continued fever alone is contagious." (p. 3.)

The same opinions had been previously adopted by Hoffmann, in his account of the epidemic dysentery of 1726. He distinguishes three forms —1st. Benign attacks without fever. 2d. Attacks with attendant fever, either quartan, or continued, or semitertian. 3d. Attacks attended by a most dangerous petechial fever, and this compound disease was highly contagious. Later writers have uniformly distinguished the simple and the complicated dysentery, and among these we may particularly specify Dr. Copland, who, in his elaborate article, has given us an admirable epitome of the accounts of writers, elucidated by his own experience of the disease. In the remarks we have to make, we shall follow the same order, discussing first the simple dysentery, and secondly, its combinations.

### I.—SIMPLE A-PYRECTIC NON-CONTAGIOUS DYSENTERY.

The greater part of the difficulties connected with the inquiry as to what is "simple dysentery," arise from the imperfect study of the morbid anatomy of the disease by the older writers. Even Morgagni has left very few dissections of dysentery, as he declined to dissect any who died of the epidemic.* Pringle gives only five detailed dissections in his

* We are inclined to regard the opinions of Morgagni in a somewhat different light than some late writers on dysentery have done. Morgagni did not from observation deny ulceration in dysentery; on the contrary, he admits its frequency. In the 31st Epistle he writes thus: "Whether there are ulcers in the large or in the small intestines, it sufficiently appears from all these observations that the intestines were really ulcerated in those dysenteric bodies from whom they were taken." (He is referring to cases detailed by Brunnerus, Valsalva, Platerus, Bossius, Fantonus, Panarolus, &c.) "Yet," he goes on to say, "in those dysenteric cases whom we have it not in our power to dissect are we also to suppose ulcerations for this reason, that, as Celsus says, they have discharged some kind of mucous portion with blood and sometimes with portions of flesh, as it were? it is worth while accurately to consider this question." He argues this question at considerable length, and seems to agree with Sennertus, who regarded these excreted membranes as mere mucous concretions, using the term "mucus" in a loose sense, to express any effusion, even of the blood. Morgagni, however, states with Tulpius, that true membrane is sometimes discharged. He sums up the argument thus: "It is more than sufficiently shown above, that those bodies which are

'Diseases of the Camp and Army,' and Dr. Hunter informs us that Pringle told him he placed little reliance on any of the dissections made in the military hospitals. Durandeau, whose work contains a most learned discussion on the causes and symptoms of epidemic dysentery, knew so little of its morbid anatomy, that he believed the seat of the disease to be chiefly in the mesentery.* Of late years, however, the morbid anatomy of dysentery, both in tropical and temperate climates, has been studied with considerable care, and in speaking of the simple dysentery we shall confine ourselves almost entirely to this single topic. The admirable lectures of Dr. Baly, who has had the opportunity of extensively observing dysentery in the prison at Milbank, contain an able discussion on the morbid anatomy, and the attempt made in them to include in one general expression all the forms described by writers is particularly ingenious and interesting. If it should appear that this attempt has not quite succeeded, or rather if, after much consideration on this subject, we hesitate to accept Dr. Baly's expression of the morbid changes, in all its extent, we do so with the admission that he has introduced into the subject a new point of discussion, and has brought into a form which admits of an accurate and defined examination the chief post-mortem appearances ascribed to dysentery. Into this examination, we now purpose to enter as fully as our limits will admit.

We must, however, first allude to the chief differences in the accounts of observers at the present time. It is almost unnecessary to say, that all agree in considering dysentery to be a disease chiefly, and in some forms, entirely of the large intestine, and, in the first instance, of its mucous coat.

*Healthy structure of the mucous membrane of the large intestine.* We may briefly remind our readers that the elements of this compound mucous coat are as follows: 1st. A basement or proper membrane which doubles upon itself and forms the tubular follicles, whose orifices are seen when the surface is examined with a lens. 2d. A columnar epithelium lining all the convolutions of the membrana propria. 3d. A subjacent vascular layer containing the nutrient vessels, between the meshes of which lie cytoblasts and amorphous matter; and, 4th, certain bodies situated in the substance of the membrana propria, or even below this, and called the "solitary glands." The solitary glands, the basement membrane, and the epithelium are all extravascular, and derive their nourishment from the vascular layer beneath. The solitary glands are stated by Dr. Baly to be sometimes open and sometimes shut; to have thick walls not composed of a simple membrane, and to contain granular matter. Like the glands of the small intestine, these glands are comparatively much larger in the fœtus and in young children than in adults or in old people. In the fœtus Dr. Allen Thompson described them as simple closed vesicles. This appearance we have seen in the colon of a middle-aged man who died of Bright's disease, and who had taken elaterium before death. The glands resembled exactly the vesicles of varicella.

discharged by dysenteric patients in the form of membranes are no proof of the intestines being ulcerated, unless they are real membranes." He then asserts the same thing as regards the fleshy excrescences, and concludes that mere discharge of blood is no proof of abrasion of the mucous membrane, and he argues this point from analogical reasoning, derived from the stomach, uterus, &c. He follows, in fact, the same train of reasoning which has been used by many later writers on hemorrhage.

* Traité de la Dysenterie, vol. i, p. 173. Bruxelles, 1789.

In some cases it has appeared to us that the tubular orifices can be traced over these glands; at other times, they seem only partly to cover them, becoming shortened apparently as they pass towards the centre of the gland, till they cease altogether. When the glands are much enlarged, however, the follicles are seen round, but decidedly not on them.

*Morbid Anatomy.* Inflammation of the solitary glands has been considered by some late writers as the first morbid condition in dysentery, and the existence of these bodies only in the large intestines has been considered to be the cause of the limitation of the disease to these parts, except in long-continued or complicated cases. On the other hand, several eminent anatomists describe the process as always one of rapid, and, in the first instance, superficial inflammation, leading inevitably and speedily to mortification, and unattended by any special disease of the solitary glands. At least, we know not how else we can interpret the description given by Rokitansky, who states that even in the first or slightest variety, the mucous membrane is swollen and red, and may be removed in the form of a pulp from beneath the furfuraceous and vesicular epithelium; while in the after stages, and in the severer varieties, the mucous membrane is transformed into a gelatinous and easily separable substance; or is in the worst form in a state of complete sphacelus, black, friable, and offensive. Lebert's description sufficiently agrees with this to prove that he has observed the same pathological state. (Physiologie Pathologique, par H. Lebert, tome i, p. 216. Paris, 1845.) Between these two extremes we should perhaps place the description given by Dr. Morehead, which, as few probably of our readers have seen, we will give more in detail.

The morbid appearances of the mucous membrane are arranged by Dr. Morehead under four heads:

1. Changes in colour and texture of the membrane. The changes in colour are described as witnessed chiefly in chronic dysentery, when there is a dark, red, gray, or black discoloration, with softening or more generally thickening of the mucous membrane. The changes in texture consist in enlargement of the solitary glands. On this point Dr. Morehead remarks:

"Under circumstances of increased secretion the mucous follicles [the solitary glands are implied by this term] of the colon become more or less prominent, and their orifices very distinct. Under these circumstances the enlargement of the mucous follicles is as yet unaccompanied by inflammatory action, but there can be no doubt that this action very quickly succeeds, and is marked by a circle of vascularity attended with softening, surrounding the orifices of the follicles, associated in some cases with thickening of the mucous membrane, in others with ulceration." (p. 139.)

2. Effusion of granules of lymph in the colon, or in the small intestines in chronic dysentery, in persons of cachectic constitutions. The effusion of lymph for some extent over the surface of the mucous membrane in shreds or tubular portions. Dr. Morehead observes that this "is a morbid lesion which takes place, but," he adds, "I do not think it can be frequent in tropical dysentery. In the cases reported by me, I find it present only in one, and that was not a case of dysentery." (Op. cit., p. 140.)

3. Ulcers of the large intestines; these are transverse or circular.

"The first form, either in separate bands, or in several bands coalescing, is generally found after acute attacks of dysentery, and most commonly is associated with more or less thickening of the walls of the intestine. The appearance of the ulcer varies according to the circumstances of stage, state of the contiguous tissues, and condition of the constitution of the subject of the disease. The bed of the ulcer may be occupied with a slough, or the slough having been thrown off, the muscular coat may be exposed, and the edges of the ulcer be irregular and thickened; or the surrounding mucous tissue may be in a more healthy state in itself and in its relation to the subjacent tissues, and the ulcer may show a tendency to cicatrize." (p. 141.)

This form of ulceration is produced, according to Dr. Morehead, by effusion of serum and lymph in the subcellular tissue of the transverse folds: "Serum and lymph become effused into the subcellular tissue, and the relation of the mucous coat to those tissues on which its nutrition depends being thus altered, its vitality is lowered, and a predisposition to ulceration is created. The circular ulcers plainly originate in the mucous follicles." (p. 141.)

4. The separation of the mucous membrane in shreds or tubular portions. Dr. Morehead formerly doubted the occurrence of such separation of the mucous coat, but is now satisfied that it does occur, and attributes it to diffuse inflammation of the submucous cellular tissue, "analogous to the diffuse inflammation of the subcutaneous cellular tissue."

The lesions of the other coats of the intestine are stated to be thickening from "effusion of lymph into the submucous cellular tissue;" and from the general impairment of the nutritive process thence resulting, is produced "the easily lacerable condition not unfrequently found after death in the worst forms of the disease."

Combining together the several accounts of these and other recent writers on acute dysentery, and assuming, for the sake of the argument, that they all belong to the same form of disease, we find the chief changes in the large intestines to be an affection of a special structure, viz. the solitary glands, and ulceration commencing in these bodies; ulceration produced by effusion of serum, lymph, or pus beneath the mucous membrane; general thickening of all the coats of the intestine from effusion of lymph and hypertrophy of the muscular fibres; an acute inflammation of the general mucous membrane, producing even in the slighter form a failure in nutrition, as indicated by the pulpy state of the mucous membrane, and leading rapidly to the production of sphacelus.

All these forms have been met with by Dr. Baly, in the prison at Milbank. In the slightest or most elementary cases of dysentery, he found the solitary glands enlarged, red, and sometimes disorganized on the summits; the mucous membrane round them was also rough, and covered with an aphthous layer composed of epithelium, mixed with an amorphous matter, probably fibrin; in forms more severe, the changes were still most marked in the solitary glands, which had lost their vitality, and were converted into small sloughs; in addition, the mucous membrane in which they were imbedded was inflamed and thickened, and likewise altered on its surface; this alteration of the intermediate mucous membrane seemed to consist in a loss of vitality of part of the membrana propria; in one case these sloughs of the membrana propria formed "strips of a rough substance, which might be taken for portions of false membrane, or fibrinous exudation," but on examination with a lens, the orifices of the tubular follicles were seen on these patches. In some cases this loss of vitality in the intermediate mucous membrane, was equally prominent with the affection of the solitary glands, so that these bodies had evidently perished simultaneously with the adjacent mucous membrane, and not previously. These gangrenous portions, implicating all the elements of the mucous membrane, were generally situated on the transverse folds or plicæ. In the third or most severe form of dysentery, the same process of sphacelation was found in its most intense degree; the mucous membrane was swollen and dark red; the submucous tissue was tumid and formed prominent masses; crusts of disorganized epithelium and lymph lay on the diseased surface, and rapidly dark sloughs were formed, involving the whole thickness of the inner coat.

It is Dr. Baly's opinion that all these appearances are to be referred to the same disease; that the special affection of the solitary glands, and the general sloughing of the whole mucous membrane are only degrees of the same action; and that the solutions of continuity in the mucous membrane, of whatever size and shape they may be, are all derived from the same process, viz. one of mortification and sloughing.

In acute dysentery, according to this view, the earliest changes occur in the vascular layer, which is subjacent to the extravascular basement membrane, glands, and epithelium. This tissue being attacked with inflammation, the blood stagnates and coagulates in the capillaries; thus deprived of nourishment the extravascular parts perish, and are thrown off as sloughs; if the inflammation be moderate, the solitary glands chiefly suffer, because they are the most destitute of vessels. "The glands lose their vitality, and together with the portion of surrounding tissue in which the circulation has ceased, are thrown off as small sloughs." (Baly, p. 11.)

If, however, the inflammation be more severe, the glands only suffer in common with the other extravascular parts, and are thrown off in common with them; thus are formed the larger ulcers in the severer forms. An ulcer once formed by the detachment of a slough may, however, Dr. Baly thinks, increase in size and depth by true ulcerative absorbtion, but this chiefly occurs in cases when the inflammation has become inactive.

"It is probable, that when the inflammation is no longer active, the mucous membrane dies in small portions, and that these dead particles of tissue are wholly, or in great part, destroyed by the fluid exudation, and not cast off as solid sloughs." (p. 11.)

This is, however, according to Dr. Baly, only an after occurrence; and the proper expression of the process is, that it is one only of sloughing.

The difference between this mode of regarding dysentery and that entertained by some other writers is greater than at first sight appears. For instance, we will take the anatomical appearances as detailed by Dr. Parkes, whose work we reviewed in a late Number. According to this writer, the anatomical signs of true dysentery are primarily derived from inflammation of a peculiar structure, the solitary glands of the large intestine; the first effect is an enlargement of the glands, and if the inflammation continue unabated, ulceration speedily sets in; if the inflammation still remain unchecked, the ulceration, after destroying the gland, spreads into the general mucous membrane, and afterwards implicates all the coat of the intestine.

"The inflammation spreads from the glands into the surrounding membrane, and then implicates the muscular, and it may be, the peritoneal coats. Lymph is in this case effused copiously between and among the different tunics, and upon the rapidly extending ulcers, forming irregular, gelatinous, whitish or dark-coloured projections and masses; the whole gut may be covered in this way, and the ulcers are then disposed to become gangrenous."*

After the inflammation has extended to the submucous coat, according to Dr. Parkes, ulcers may be formed by effusion under the mucous membrane which is then raised in spaces of greater or less extent, and finally detached. In this way, he thinks, are thrown off in all probability those portions of mucous membrane of greater or less size which are occasionally discharged. This result, however, is regarded by him as only an epiphenomenon, and consequent on the true dysenteric process.

The chief peculiarity of this description is the prominence given to the special affection of the solitary glands; this is declared to be the elementary and essential change, although general inflammation, sloughing, or effusion of fluid into the submucous cellular tissue may follow in severe attacks. If, however, it could be proved that ulceration occurred on the transverse plicæ, or in other parts, or that there was general sloughing, without any particular development of the solitary glands at the commencement of the case, then it follows, either, that these are not cases of common uncomplicated dysentery, or that the description above given is partial and incomplete.

In Dr. Baly's opinion, the solitary glands are only primarily diseased, because they are the most extravascular parts; at least this is a corollary from his doctrine of the process being always one of sloughing; when the inflammation is intense, the other parts of the membrane suffer simultaneously, and sloughs are formed without any special disease of the glands. In the severer forms, and not in the mildest, the true type of dysentery is seen, and to explain the elementary steps of the process, it is necessary to consider chiefly the advanced phases.

It is true, however, that this is not the view formally proposed by Dr. Baly, who, in several places, distinctly intimates, that the solitary glands are primarily implicated, and in his third lecture, writes as follows: "In dysentery as well as in fever, the intestinal lesion is the effect of a chemical action taking place between the glands or the tissue immediately surrounding them, and a morbific matter circulating with the blood." (p. 28.)

Possibly then, unless we are interpreting Dr. Baly's opinion erroneously, this doctrine of sloughing does not, when critically examined, fully express Dr. Baly's own conviction of the part which the solitary glands play in the dysenteric process.

But it is necessary to examine this view a little more minutely; we have considered it as a generalization; perhaps we might almost say an hypothetical generalization. Dr. Baly has attempted to define a process which should satisfactorily account both for those cases in which the solitary glands are wholly or chiefly affected, and for those in which the general mucous membrane perishes without any special superabundant affection of a particular structure. Does this generalization then explain all the phenomena? are all the shades of morbid changes explicable by its aid? and is the classification of these changes, and of the symptoms which announce them, simplified by its application?

The questions we have to ask, are, whether there are proofs that the solitary glands perish, and are thrown off as solid sloughs; or, supposing

* Remarks on the Dysentery and Hepatitis of India. By E. A. Parkes, M.B. Lond. 1846, p. 13.

the solution of continuity to be thus formed in the first instance, is it a fact that the further progress of such solution of continuity occurs by a repetition of the same process, and not by true ulcerative absorbtion?

It appears to us that either of these positions requires more evidence to prove it than we find in Dr. Baly's lectures. It is true, he describes the summits of the solitary glands as occasionally presenting marks of disorganization or minute yellowish sloughs; but he gives no series of cases proving the existence of the same process in the case of the whole gland, as well as of a part of it, or of the tissue round it; and he details no gradual transition into those cases in which the general mucous membrane perishes.

If we consult authors who describe the commencement of dysentery with sufficient minuteness, we find that they never appear to have met with cases in which the whole solitary gland sloughed out, but to have found the gland enlarged, and then ulcerated at the apex in a minute point. This is nearly the description given by Dr. Hunter,* in his work on the Diseases of Jamaica, and his description is so clear and accurate, that although it has often been quoted, we shall make no apology for again inserting it.

"Upon a first view," says Dr. Hunter, in speaking of the morbid anatomy of dysentery, "the bowels, particularly the colon, appear irregularly contracted, and redder than natural at the contracted part. On cutting out portions of the gut and examining the internal coats, the appearances of disease become more evident. There are to be seen small tubercles like pustules, sometimes in a smaller, sometimes in a greater number; and *they are to be found in different stages, so that their progress can only be collected from several observations combined.* The same subject will frequently furnish in different portions of the gut examples of the several stages. Their progress appears to be nearly as follows: there is first, a small round tubercle of a reddish colour, and not more than 1-10th of an inch in diameter; it increases gradually till it be near a quarter of an inch in diameter, and becomes paler as it grows larger. In this stage, there appears a slight crack on the top with a slight depression, which gradually increases, and on examining the contents of the little tumour, I have generally found them to be of a cheese-like substance. The pustule, for though it contains no pus, I do not know any name more expressive of its appearance, is seated under the villous coat, between that and the muscular coat. As the opening enlarges, the edge becomes prominent, and the base becomes rough and scabrous, from which matter oozes out that is sometimes tinged with blood. Such is the progress of one, but they are often in clusters, and become confluent so as to form a rough, unequal, ulcerated surface, with a hard and thickened base. These morbid appearances probably take place, more or less, in all cases of epidemic dysentery."†

Hunter afterwards remarks: "Sir John Pringle has indeed mentioned mortifications, gangrene, and abrasions of the villous coat, none of which I have ever seen." (Op. cit. p. 184.)

In this description it will be observed that a special affection, evidently of the solitary glands, is described, and the intermediate mucous membrane is left altogether unnoticed. Considerable importance also is attached to

* On the Diseases of the Army in Jamaica. By John Hunter, M.D. London, 1796. p. 184.

† Morgagni had evidently seen the glands enlarged in chronic dysentery and diarrhea. In the 65th Epistle he speaks of the case of an old woman worn out by diarrhea. "The rectum was almost universally livid in consequence of inflammation, and was here and there tumid internally, but especially at the lower part, where a circular patch, extending to the extent of a man's thumb, was soft and prominent, and above this patch were protuberant, either true lenticular glands, or glands similar to them, lying at a distance from each other, reddish and brown in colour."

the fact, which Dr. Baly has passed over almost without notice, that great enlargement of the glands precedes in many cases any further change. These enlarged glands are not then thrown off, but a "crack forms on the summit," which slowly increases. Now whether this increases by true ulcerative absorbtion, or by some mode more analogous to sloughing, but of which we have not any very clear idea, is difficult to determine, and is perhaps unimportant. The great matter is that there are no "solid sloughs," or at least none were noticed by Dr. Hunter.

Dr. Parkes describes the early changes in the glands, without seeming aware that solutions of continuity are produced by their entire destruction and detachment.

"If the inflammation be slight," he says, "the glands acquire a considerable size, without any ulceration, and when this does occur it is chiefly at the apex, and the thickened contents of the gland are not immediately discharged; in the more acute cases, the glands are suddenly increased in size, and press equally upon the mucous membrane covering them, thus causing its uniform destruction, and the immediate discharge of the glandular secretion."*

Now in the case of these two writers, it is hardly conceivable that they should not have noticed the state of the surrounding membrane, if it had been in that stage of engorgement, which Dr. Baly's description would lead us to consider necessary, in order that the supply of nourishment to the extravascular solitary gland might be suspended.

Dr. Parkes proceeds further. In the very next paragraph to that we have extracted, he says:

"In the majority of instances, the intermediate mucous membrane is little affected until ulceration has advanced to some extent. The exceptions to this rule occur in those cases generally complicated with gastro-enteritis, or with scurvy, in which there is a superficial erysipelatous inflammation implicating the whole of the mucous membrane."†

These descriptions refer only to the commencing and slight cases; let us take another case in which sloughing was really going on. In his 31st livraison, Cruveilhier gives a plate of what we, as well as Dr. Baly, consider to have been a case of dysentery. Numerous sloughs are depicted of various sizes, but the majority are roundish, and in several places the sloughs are quite round and minute, being evidently produced by the destruction of only one or two solitary glands. Now in this case, which, Cruveilhier remarks, merits the name of Dothinenterite, the intermediate mucous membrane was perfectly sound. "Du reste, je ferai remarquer l'integrité parfaite de la membrane muqueuse dans l'intervalle des eschars."‡

Dr. Baly cites this case as an example of the diffuse gangrenous inflammation, but this "perfect soundness" of the intermediate mucous membrane seems to us to be irreconcilable with such an opinion. We should rather be disposed to believe that the gangrenous condition of the inflamed solitary glands was owing to a particular constitutional taint or disposition, for in the same case (one of puerperal fever) there was pus in the uterine veins, and erysipelas of the surface.

If we sum up our argument so far, it will be seen that the points most adverse to Dr. Baly's description are—1st, the enlargement of the glands,

* Op. cit., pp. 8-9. † Op. cit., p. 9. ‡ Cruveilhier, op. cit.

which mere stagnation in the surrounding capillaries could not accomplish; 2d, the perfect state of the intermediate mucous membrane in many cases, which membrane, as far as the mere hyperæmia or collection of blood is concerned, ought to be as deeply involved in the process as the solitary glands; and 3d, the total want of evidence that the glands are, in all cases, thrown off as sloughs.

Although we doubt, therefore, whether the whole gland is ever thus at once detached, there can be little doubt, that occasionally the first solution of continuity is produced by a small slough at the summit of the gland, as described by Dr. Baly. The enlarged gland, with its small slough, presents so great a resemblance in such a case to the smallpox pustule, that almost all authors have used it as a term of comparison. Thus Sir John Pringle, in his 5th dissection, describes protuberances of a roundish figure, and adds:

"We all agreed that we had never seen anything so nearly resemble the smallpox; they differed from variolous pustules in this, that as far as we examined them, they were of a firm consistence without any cavity. Mr. Hewson told us that he believed they took their rise from the cellular membrane which lies immediately above the villous coat, for that some days before, having opened another person who had died of the dysentery, he found the appearances much the same as in this subject, and particularly with regard to these tubercles which he had examined at more leisure. These protuberances were only in the larger intestines, for though we likewise opened the smaller, we could observe nothing similar to them there.*

It must have been a similar state of the glands which led Nyander to remark that: "Dysenteria epidemica scabies est intestinorum interna, ut dissectionibus cadaverum, Dissenteria defunctorum patet."†

Zimmermann‡ says: "Sometimes small pustules have been seen in the bowels, and in the intestina crassa little aphthæ, that bled when pressed, and looked like the flat kind of smallpox, only they are solid and without any cavities."

Chisholm, Ballingall, and a host of subsequent writers have also made similar remarks respecting the resemblance to the smallpox.

We do not think, however, that it is a fair induction to conclude that the process is always the same even in this respect; in many cases the dark spot on the summit, whether slough or orifice, which gives the resemblance to the variolous pustule is wanting, and the first solution of continuity appears as a minute ulcer; thus Dr. Parkes writes of one case: "All the stages could be seen here; there were glands distended with white substance, ulceration of these at their points, then destruction of glands, and production of small ulcers with raised edges, then the edges flattening as the ulcer increased in size, then the usual ulcers of all sizes." And again he writes of another case: "Some of the glands were a little reddened and hard, others were redder and harder; others had a minute orifice at the apex, and others were completely converted into small ulcers."

If these descriptions are correct, we do not see how it can be doubted

* On Diseases of the Camp and Army. 5th edition, 1765. p. 246.

† A Treatise on the Dysentery, with a Description of the Epidemic Dysentery of Switzerland in 1765. Translated from the original German of J. G. Zimmermann, M. D., by E. W. Hopson. London, 1781. p. 35.

‡ Amœnit. Acad., vol. v, Dissertat. lxxxii, p. 97.

that the glands in these cases were destroyed, either by true ulcerative absorbtion, or by a process indistinguishable from this.

Moreover, Dr. Baly so far qualifies his position, as to allow that the ulcers may increase "by a solution of the tissues" when the inflammation is subdued in severity. How can we determine the frequency of this occurrence, and decide when ulceration or sloughing is going on; or if ulcers will increase in this way, how are we to feel certain that when the inflammation is moderate, they may not even commence through the agency of the same process? And even when the inflammation is intense, the appearance which a fully-formed ulcer presents, with its sharp, jagged, overhanging, and vascular edges, here and there eaten away like a common active, cutaneous ulcer, is much more like what we have been accustomed to refer to true ulceration than to sphacelus.

We must now proceed to notice, very briefly, some of the more severe varieties of dysentery.

It is a fact, admitted by all, that there is a form of disease of the large intestine, characterised by the inflammation being intense, and leading rapidly to gangrene. Rokitansky's description applies more particularly to this form, and Dr. Baly relates a well-marked case "in a feeble young man, who, when he had been two years in prison, was attacked with dysentery, and died after nine days' illness." The other case related by Dr. Baly was one complicated with typhus, and therefore should be excluded from examination.

The great peculiarity about this disease is that, as a general rule, the solitary glands are not peculiarly and primarily diseased. Occasionally, however, the special affection of the glands is conjoined with the diffuse gangrenous inflammation. The lower part of the ileum is also more frequently diseased in this form, on which account Chisholm was disposed to believe there were two varieties of dysentery, one confined to the large intestines, the other extending to the ileum.

The absence of all affection of the glands in some of these cases is of course attributable, according to Dr. Baly's hypothesis, to "complete stagnation of the circulation and gangrene of the tissue;" the consequence being, that "no small points indicating the situation of the affected glands are seen, all these are lost in the extensive and inflammatory congestion and mortification."*

But it is evident that although this complete destruction may involve the intermediate membrane as well as the glands in the parts chiefly affected, it is highly improbable that at some parts the disease, in its slighter and more elementary shape, should not show itself, and that the several stages should not be seen in different parts of the intestine. To this obvious objection, Dr. Baly replies as follows:

"This hypothesis," he says, "describes correctly the kind of inflammation which exists in the cases of dysentery here referred to, but it may appear not quite satisfactorily to account for the total absence of all enlarged and solitary glands on those parts of the mucous membrane of the colon, which are only in the state of inflammatory congestion; such as are seen in some parts of these drawings. To explain this, however, I would suggest that possibly only the inflammation set up by the primary cause of the disease has the tendency to produce especial tumefaction and mortification of these glands, and that inflammation secondarily excited

* Op. cit., p. 487.

does not affect them more than other parts of the mucous membrane. Assuming this, we may explain the cases in question by saying, that in these instances all the glands attacked by the primary cause of the disease are involved in the gangrenous parts, while the inflammation in the portions which are not disorganized has arisen secondarily by sympathy with the neighbouring intensely affected portions, and not from the direct operation of the original cause of the disease; and that on this account the glands are not apparently affected."*

Now we must say that this explanation does not at all satisfy us. Are we, for the sake of an hypothesis, to assume that the inflammatory congestion and the diffuse sloughing, which are surely but degrees of the same process, are produced by two different causes? Are we to distinguish two diseases in the sloughing dysentery, one the real affection, and the other its consequence? Dr. Baly himself seems to admit the weakness of his position, for he immediately proceeds to adduce another reason:

"It should be borne in mind, too, that the solitary glands of the large intestine are not at all times in the same state of development, and that when dysentery attacks them in their lowest condition, as to size and activity of function, they will, in all probability, show themselves less conspicuously in the inflamed mucous membrane."

Put this explanation to the test; suppose an intestine, whose solitary glands are at their minimum of size, to be attacked with dysentery. What is the consequence? Why, that the solitary glands would become immediately enlarged, as activity of function is the earliest change in the disease. And if we could suppose in the severest forms, a part to die so rapidly that the solitary glands had not time to fill with contents, we could not imagine any similar cause to prevent the development of the glands on the less affected portions of the mucous membrane. In these portions why should we not see, as Dr. Baly expresses it, "the distinctive character usually given by the especial activity of the diseased process in these glands?"

Are we then to conclude that the diffuse gangrenous form is a different disease from that in which the solitary glands are primarily and principally affected? Dr. Baly remarks on this point:

"The difference of anatomical characters in different cases is sufficiently great to accord with such a view; if the affections of other parts complicating the disease in the large intestine, if the symptoms and the influences causing the disease in the respective cases, should present well-marked differences." (p. 437.)

After an examination of these several circumstances, Dr. Baly decides that the two forms are the same disease, differing only in degree, and the difference between them is only like that existing between the malignant and the benign form of scarlatina.

Our space will not permit us to enter into the examination of this question, but before finally leaving it we wish to direct the attention of inquirers to a phenomenon which has not yet been sufficiently studied. We have witnessed several times in dysentery, and in other cases in which there was dark inflammatory congestion of the mucous membrane tending towards diffuse sphacelus, roughness of the mucous membrane with effusion of granular lymph upon it. This lymph has been in many cases evidently effused over the course of the larger vessels, and it has generally occupied the summits of the plicæ. Although it has often been effused upon the mucous coat, in some instances it has seemed to press on and destroy this coat, and then by its disintegration and detachment to form apparently sloughing ulcers. We would recommend attention to this phenomenon, and to the condition of the larger vessels in these and similar cases. It may be that the state either of the vessels or of the blood has an effect on the type of the inflammation. But it is our part to probe and examine the views of others, and not to intrude our own, and we must now quit this portion of our subject with the expression of our hope, that we may have occasion at no long period to notice a further discussion on dysentery from the able pen of Dr. Baly. We doubt not that he will be able to explain his opinions, and to adduce his evidence with greater minuteness than the short space of the Gulstonian lectures has allowed him time for. We have criticised his description in no unkind or captious spirit, but simply with a desire to arrive at truth and certainty. We are sure Dr. Baly will agree with us when we say, that in dysentery, as in all other diseases, much remains to be examined and to be understood, and that the vantage ground we have gained, and the fresh light we boast, show us not the end of our labours, but a fresh country and an unexplored and untrodden soil. If no writer has yet given the full expression of the morbid anatomy of this disease, we must attribute it to the necessary imperfections of our science and to the inherent difficulties of the subject. But the advances lately made by Drs. Baly, Morehead, and others encourage us to hope that our progress will still be uninterrupted; if difficulties have arisen, we can recur to that great book of Nature in which lies the solution of our doubts and hesitations; and, as has been beautifully remarked by an eloquent writer on a very different subject, like the engraver, who, cutting deeply into his gem, trieth the impression upon the tender wax, and finding the lines not deep enough, is not disheartened, but with earnest labour returneth again to his pleasing task, so we, finding our definitions too narrow, and our descriptions too restricted, must return again to the great teacher, and with humble and attentive eyes, scrutinize anew the knowledge upon which she permits us to gaze.

### II.—COMBINED AND COMPLICATED DYSENTERY.

The combinations of diseases are deserving of the closest study, and constitute indeed some of the most important as well as difficult points in medicine. This combination may be of different kinds; there may be mere accidental co-existence, with or without temporary modifications of symptoms and manifestations. Or the relation may be of a more intimate kind; diseases may arise from the same cause, or may act as causes to each other, or may produce that condition of body in which a previously latent cause becomes operative. To prove, however, this last intimate connexion between diseases requires no little care, and no slight amount of evidence. Even when the great number of times in which one disease follows another, proves that there is something more than accidental concurrence, the successive steps of the production of the secondary disease can at present be seldom determined. As certain diseases seem thus to compound themselves, so also some seem rather to antagonize and nullify each other, and of these "incompatible diseases," lists have been given by Rokitansky and others. But here greater caution even is necessary than in the former case, and conclusions should be deduced only from a most extended series of numbers, otherwise errors result from limited observation, and from the occasional absence of the cause of the presumed incompatible disease. For example, Rokitansky asserts the incompatibility of dysentery and typhus, yet not only is this combination very common, but the express purpose of one of the works before us is to prove that the union of the two diseases gives its contagious character to dysentery.

The chief diseases with which dysentery complicates itself, in the proper sense of the term, are intermittent, remittent, and continued fevers. In these cases the active cause apparently produces both diseases. In other cases, according to some authors, dysentery tends to the production of other diseases; the most prominent of these are an affection of the liver in some shape, and less commonly, diseases of the kidneys, pancreas, spleen, bladder, or small intestines. The diseases with which dysentery occasionally allies itself by some nearer link than that of mere co-existence are rheumatism, erysipelas, scurvy, or purpura, delirium tremens, peritonitis, and phthisis. In all other cases the prevalence of dysentery with another disease is accidental.

The most important fact which results from the true complications of dysentery, is that they cause modifications either in the symptoms or in the post-mortem signs of the disease. Thus in one variety attended with remittent fever, the stools are not truly dysenteric, but are watery, yellowish, often without blood, while the anatomical signs in the large intestine remain the same; in the scorbutic variety the post-mortem appearances are changed, sloughing is more common, and the ileum participates constantly in the disease. A peculiar adynamic type is also often impressed on dysentery by malarious fevers, on which account several writers have assumed that uncomplicated adynamic dysentery has no existence, but that the lowered type is attributable always to the attendant fever. This is opposed, however, to the experience of other observers, and particularly of Dr. Copland, to whose views on this point we shall have occasion presently to allude.

The combinations which we shall now discuss are, 1st, with malarious fevers; 2d, with typhus; 3d, with diseases of the other abdominal viscera.

1. *With malarious fevers.* So common is the union between remittent fever in all its forms and dysentery, and so evidently are both diseases excited by the same cause, that some writers, as Williams, Bancroft, &c., have believed dysentery to be always, or in the majority of instances, of malarious origin. But this is perhaps an extreme opinion, since dysentery is often found to prevail without a single case of ague being seen at the same time. In temperate climates this complication was formerly much more common than it is now, and many of the recorded epidemics must be considered to have been of this variety. Thus the epidemic dysentery, recorded by Morton, was attended by a violent remittent fever, which indeed prevailed much more extensively than the dysentery, and which, apparently from its tendency to pass into typhoid fever, he terms "febris Συνεχης." It was of this fever that Cromwell died, and it raged from 1658 for several years, being interrupted only by the great plague in 1665, before which the lesser pestilence for a time seemed to disappear. The most useful treatment in the dysentery was bark and opium, nearly the same which, with the addition of local bloodletting, we observe to be recommended by a late writer on this disease occurring in the tropics. The "febris dysenterica" of Sydenham was perhaps of the same kind. Dr. Harty remarks on this point:

"If this be not Sydenham's meaning, and this the origin of his opinion, I confess I cannot understand him, for if we take his phrase of "febris introversa" in its literal and obvious sense, the opinion he adopted must appear unfounded, there being no evidence of a "febris introversa," because in such a case the fever should disappear, the dysentery being substituted in its place; on the contrary, the fever does still exist in combination with dysentery." (p. 46.)

It did not always so exist, however, for Sydenham expressly mentions the occasional absence of all fever, and by his "febris introversa" we should rather infer that he intended merely to intimate that the active cause of the fever evidently produced occasionally dysentery and not fever, or, as Sir G. Blane remarks, that the two diseases were vicarious. (p. 449.) And later observers have noticed that in the same individuals dysentery and remittent fever will alternate.*

Hoffman describes two epidemics in 1719 and 1726; the first of these prevailed chiefly in low marshy places, and was associated with intermittents of the semitertian type, consequently it is referred "to some malignant miasma formed by the peculiar effluvia of the earth." The second epidemic was totally different, and did not prevail much in marshy places, but raged more in hilly and elevated spots; it was also sometimes contagious. The "putrid dysentery" of Bengal, described by Clarke, was in all probability this complicated form, or a combination of this with scurvy. Pringle noticed that exposure to the same cause produced marsh fevers and dysentery alone or in combination.†

Perhaps one of the most instructive examples of the combination is given by Roederer.‡ Intermittent fevers and dysentery were epidemic, in one row of houses, he proceeds to say, the epidemic intermittent fever raged alone; in another row, situated on higher ground, the dysentery alone raged, and destroyed many; while in a third row, situated between the two former, both diseases were very rare. (p. 31.) Of late nearly every writer on dysentery has noticed its combination with malarious fevers, in almost all parts of the world; and Jamaica, India, west coast of Africa, Walcheren, and the Low Countries generally, and some parts of Spain and Portugal, are the places which have appeared chiefly subject to it. The chief conclusions to which we arrive by a study of this combination, as far as it is known, are these: 1st, that marsh fevers and dysentery often unite in the same individual; 2d, that when they appear in different individuals, they yet often testify, by their simultaneous increase or decrease, the presence of a common cause; 3d, that they may supersede each other in some cases, or may alternate with each other in point of prevalence, as if they were produced by analogous but not identical causes.

The symptoms of dysentery in the malarious variety, either combined with intermittent or remittent fevers, or separate from these, present several varieties: 1st, symptoms of true bloody flux—dysenteria cruenta; 2d,

* Buel on Fever in Massachusetts; Hargreave on the Walcheren Fever.

† Cleghorn says, "A tertian is sometimes changed into a dysentery, or the converse." (Diseases of Minorca. 4th edition, 1779, p. 134.)

‡ Tractatus de Morbo Mucoso, J. G. Roederei et Car. G. Wagleri. Edited by G. H. A. Wrisberg. Gott. 1783.

symptoms more approaching to cholera, viz. loose, watery, copious stools, without much tenesmus—approaching to the dysenteria incruenta of Willis; these two forms are generally attended with manifest remittent or intermittent fever; 3d, adynamic symptoms, with loose, bloody, and grumous stools, and rapid failure of strength: a remittent fever often accompanies this form also, but in other instances, although undoubtedly produced by malaria, there is a total absence of all fever.*

The anatomical signs in the large intestine are apparently similar to those witnessed in the simple form of the disease; or rather no differences have been yet described.† The small intestines are often diseased. The dysentery at Milbank would appear to be of malarious origin, and Dr. Baly remarks, that the description given by Davis of the anatomical appearances in the dysentery attending the Walcheren fevers is "identical with the disease from time to time observed at Milbank."‡

* Morgagni was attacked with the dysenteria incruenta, and, in his 31st Epistle, art. 11, relates his own case admirably. He was travelling through a marshy district, and towards evening began to be attacked with purging—during the night he discharged "at least sixteen pints of almost limpid water; the pains were slight, the stools not frequent, but very large." On the following day he perceived that his face and body generally were grown lank and thin, as if from an acute disease, and his mouth and fauces were excessively dry. He distinguishes this flux from dysentery, although he states that the watery and the true bloody flux are both usually comprehended under the same term. "So I remember," he says, "that epidemic fluxes of this kind were called dysenteries by the physicians of Modena when they wrote to the physicians of Bologna, which appellation the latter disapproved not of." (Epistolæ xxxi.)

† Lebert indeed describes the "dysenterie blanche" as only an irritation, "très superficielle," of the mucous membrane, with an abundant exfoliation of epithelium and a great serous secretion (op. cit., p. 216); but there is no doubt that this is true only of the very slightest forms.

‡ The account given by Davis, although an admirable description of the morbid anatomy of malarious dysentery, is true only for that form, and in his work on Walcheren Fever he gives no dissection of uncomplicated dysentery. We will extract from his work (p. 155) the references he makes to the large intestine—in many cases it is altogether unnoticed. Case 9—"Slight dysentery, rectum thickened and ulcerated, spots of effusion, and many rough, irregular, and inflamed lumps." No. 11—"Inner surface of colon covered in several places with tubercles, and in others with small ulcers resembling chancres." No. 13—"Coats of colon thickened, and inner surface of rectum black and covered with ulcers." No. 16—"Inner surface of colon slightly inflamed." No. 17—"Colon and rectum contracted, tuberculated, and ulcerated on their inner surfaces." No. 19—"Colon and rectum formed one mass of disease; their coats retained nothing of their natural appearance, neither could they be separated from one another. A vast deal of coagulable lymph had been deposited between them, and had become condensed. Their whole internal surface was completely ulcerated, and when cleared of feculent matter was found to be rugged, in consequence of some portions of the inner coat having sloughed, and of others having received an additional lining from condensed coagulable lymph. Before these intestines were washed pus exuded from numerous small ulcers spread upon their inner surface." No. 20—"Colon contracted, and had numerous small ulcerations on its inner surface, rectum slightly ulcerated." No. 21—"Caput coli as large as the stomach, being distended with air, slightly inflamed on its internal surface, which had also a granulated appearance in several spots; colon contracted, and having transverse lines with small red eminences." No. 23—"Colon lessened in diameter, and bore marks of recent ulceration; the rectum had been ulcerated, but was healed." No. 24—"Rectum and colon completely gangrenous, of a dark colour on their inner and outer surfaces, and excessively offensive." No. 25—"Transverse arch of the colon inflamed on its inner surface." No. 28—"The colon was contracted, and had red stripes round it, like rings, upon which were numerous red eminences, of the size of a pin's head, placed in rows." No. 29—"Colon ulcerated, rectum much ulcerated, and had sloughed near its termination." No. 30—"Colon and rectum had numerous tubercles and ulcerations, the villous coat of the rectum had sloughed extensively, and was so much altered everywhere as to be scraped away with the slightest effort." No. 35—"Colon ulcerated within to the extent of three or four inches." No. 37—"Colon was lined with ulcers and tubercles of the kind I have elsewhere described." No. 41—"Colon red and scabrous." No. 42—"Colon slightly ulcerated."

It thus appears that, in 19 cases of Walcheren fever (out of 42 recorded dissections), the large intestines were diseased from attendant dysentery. In 6 cases the intestine is merely called ulcerated, or slightly inflamed. In 9 cases appearances are described which leave no doubt that the disease of the solitary glands was very conspicuous. In four cases only is sloughing mentioned. In 2 of these cases the rectum only was sloughing; in the rest of the intestine the solitary glands were diseased. This slough was doubtless from the severity of the inflammation, and is a common occur-

The complications of other organs in malarious dysentery are so far peculiar, that the spleen is often diseased; the liver is also affected, being generally described as enlarged and engorged, or shrivelled and black.* It has also appeared to us that abscess of this organ is seldom mentioned, and it is a curious subject for inquiry whether the changes which occur in the liver in this variety of dysentery, assume some form distinct from that condition which predisposes to abscess of the organ. If this were so it would reconcile some discrepancies to which we shall presently allude.

Two questions of great importance arise in connexion with this subject:

1. Is the miasmatic agent producing this dysentery identical with the true febrile malaria? It evidently owns the same conditions, being developed in marshy and humid situations; but it is a very remarkable circumstance that ague, the index of malaria, is so often disjoined from it. Rollo, who found a remittent or intermittent fever to be almost an invariable accompaniment of the dysentery of the West Indies, believed that the former only arose from malaria, while the latter was produced by a simultaneous or prior exposure to cold and moisture. But this doctrine will, in all probability, find few supporters, and it is more reasonable to suppose that the virus of dysentery may be only analogous to the true febrile malaria, and may be capable of existing either isolated or combined with this.

2. Are there any grounds for the belief entertained by Bancroft, Williams, and others, and which seems to be gaining ground at the present day, that dysentery always arises from a specific virus of malarious origin?

"From these facts," writes Dr. Baly, "as well as from the consideration of the whole character of the disease, I infer that dysentery is always produced by a poison introduced into the system from without, and that in most instances this poison is generated by the decomposition of matters contained in the soil."

The chief arguments against this view are: 1. The prevalence of dysentery in its acute sthenic form in places where no marsh diseases are prevailing, and where there is no apparent development of malaria. 2. The immediate reference often made by patients to some chill or exposure to cold and moisture at times and places when marsh fevers are unknown. 3. The occurrence of the disease on hills and in cleared spaces, and also at sea, where bowel complaints of different kinds often occur on sudden changes of weather, and cannot reasonably be referred always to effluvia from bilge-water. 4. The evident production of the disease by other effluvia than those from marshes; as, for instance, from animal effluvia, as proved by the observations of Sir James M'Grigor, Vignes, the authors of the article Dysenterie, in the 'Dictionnaire des Sciences Méd.,' Copland, Chomel, &c. Dr. Harty writes on this point:

"I do not say that marsh effluvia alone are not competent to induce dysentery, but this I maintain, that the influence of wet and cold in its production is more cleary, satisfactorily, and universally demonstrable than that of marsh effluvia." (p. 57.)

rence in dysentery. In the 3d case (No. 19) there can be no doubt that it was one of intense inflammation of the usual kind, with great effusion of lymph and consequent gangrene, as described by many writers on tropical dysentery. The 4th case (No. 24) is the only one resembling the diffuse gangrenous inflammation, but the account is so short, consisting merely of the lines above given, that it is difficult to decide. At pages 190-191, where Dr. Davis sums up the appearances in the intestines, he describes minutely the appearance of the "tubercles" forming little ulcers, but he only cursorily mentions gangrene, saying, "These ulcers always occasioned a contraction of the gut, which was mostly inflamed round them, and, in advanced stages of the disease, gangrenous."

* M'Grigor, Ferguson, Davis, Fallot, and many others.

An attempt was lately made by Cornuel* to prove that malaria had nothing to do with the production of dysentery. He states, that while the "Basse Terre" of Guadaloupe was ravaged with dysentery in 1837 (1·078 treated—123 died, or 11·41 per cent.), the "Grande Terre," separated only by a river, was totally exempt, (p. 102). He states that, from the situation of the town on the Basse Terre, the cold winds from the mountains cause frequent and rapid transitions of temperature. In these mountains also are numerous torrents which become swollen, "blanchâtres et troublés," after the rains and pour down into the plains. The water which is drank at this time has a peculiar though not a disagreeable taste. Dysentery only appears at the places where this water is drank. At the Grande Terre there are few rivers, and they drink chiefly rain-water. Miasmatic exhalations, M. Cornuel adds, are more common at the Grande Terre, where dysentery is less frequent.

The influences here alluded to, viz. sudden transitions of temperature, and unwholesome water, have been always assigned by writers as causes of dysentery. Van Swieten even says, with reference to this point, that cold seizing the body when heated has killed more than the plague.† Even in epidemic dysentery, obviously connected with fevers of some sort, Zimmermann, Roederer, Huxham, Durandeau, Willan, and many others, have also admitted the operations of vicissitudes of temperature, in producing epidemic as well as sporadic attacks. The French and English military surgeons during the last war admitted these changes, and also animal and vegetable exhalations as the active agents. (Vignes,‡ Sir J. M'Grigor,§ Ferguson,|| and many others.)

We have not time to enter into this important subject, but we can assure our readers that the evidence in favour of both epidemic and sporadic dysentery arising from several causes, besides marsh exhalation, is incontestable, and we cannot but consider any opinion as doubtful and restricted, which regards peculiar anatomical characters as capable of production only by one particular cause or agent.

The question may be discussed in this place as to the real nature of Willis's "Dysenteria Incruenta." Willis states, that almost every autumn, but particularly in the years 1670 and 1671, two forms of dysentery prevailed. "In one the stools are watery and almost limpid, with a sudden prostration of the forces ; in the other the stools are bloody, but the powers remain tolerable." In 1670 the first species, or dysenteria incruenta, was exceedingly fatal about the autumnal equinox, invading suddenly, and being attended with great vomiting, and frequent watery stools. In twelve hours some were moribund, with a weak and minute pulse, with cold sweats, and a gasping respiration. These symptoms are so similar to cholera, that Dr. Harty concludes that there can be no doubt on this point. "The season of the year, the mode of attack, the con-

* Mémoire sur la Dysenterie observée à la Basse Terre (Guadeloupe). Par M. le Docteur Cornuel. Mémoire de la Académie Royale de Médecine, 1840.

† Stoll says, "I have never seen this disease to occur except when the patients had to reproach themselves with having been exposed to cold while sweating."

‡ Traité complet de la Dysenterie. Par P. Vignes. Paris, 1825. pp. 204 et seq.

§ Medico-Chirurg. Transactions.

|| Ferguson says, "True dysentery is the offspring of heat and moisture—of moist cold in any shape after excessive heat. Nothing that a man can ever put into him would ever give him true dysentery." (Op. cit., p. 188.)

junction of vomitings with copious stools, the debility and other effects immediately consequent, the rapid fatality of the disease, and the mode of treatment, all unite in pronouncing the disease to be cholera." (page 60.) And he quotes Hoffman, who distinguishes very accurately between cholera and dysentery, and who refers Willis's dysenteria incruenta to the former disease. Moreover, it appears that Willis did not confound his two species of dysentery, but states them to be "duas et *longe diversas* species."

It is rather singular, however, if this be true, that Willis did not call this disease "cholera," a term used constantly by all the writers of the period, and did not refer to the spasms so particularly described by Sydenham* (p. 277), in his account of the cholera of 1676. It must be remembered also that the combination of a malarious fever with dysentery sometimes alters its type, and impresses upon it more or less of a choleric character. This appears to be the case at the present day in the tropics. In speaking of this combination in India, Dr. Parkes says:—"The fever appeared always to impart a peculiar adynamic type to the dysentery; thus the non-effusion of lymph, the loose, dark, serous nature of the stools, *approaching in some cases to cholera,* looked more like a passive flux than an acute inflammation of the solitary glands." (Op. cit. p. 129.)

Now we know that at the time of the "dysenteria incruenta," malarious fevers were common, and in 1671, after an exceedingly cold winter and a very dry spring, they were so prevalent as to constitute an epidemic fever, which ravaged nearly all England, preserving an intermittent character for some time, but finally becoming remittent, and being complicated with true bloody dysentery, as well as with the dysenteria incruenta. It is possible, therefore, that this latter disease may have been a form of adynamic dysentery, in which, from some reason or other, the fever, usually developed by the common cause, was suppressed. But there is no doubt that there is some connexion or alliance between these forms of malarious dysentery, or even between intermittent and remittent fevers, and common cholera, and perhaps even the malignant Asiatic cholera. "The cholera morbus," writes Cleghorn, "is attended by a tertian," and in several places he describes very accurately this alliance of the two diseases. It would reconcile all difficulties if we could suppose the "dysenteria incruenta" to include in it a form of malarious dysentery, verging upon, and finally ending in common but severe cholera.

The disease described by Degner under the term "dysenteria biliosa contagiosa," was certainly the same, or nearly the same, as the "dysenteria incruenta" of Willis. Dr. Harty indeed considers it to have been an epidemic of true malignant cholera. There were copious discharges from the bowels, which were "flavæ aut potius subrufæ, sed sensim aut fiebant porraceæ, æruginosæ aut subcruentæ." The quantity of these was immense. "Almost the whole body was dissolved into liquid, and poured out by the intestines." There were also violent vomitings, epigastric pain, and rapid failure of the powers, testified by constant faintings, and diminution, with intermission of the pulse. He describes four cases: two deaths, each on the 8th day, and two recoveries.

The most characteristic symptoms of the Asiatic cholera, viz. the coldness and colour of the surface, the loss of voice, the cramps, the embarrassed respiration, and the *early* death, are left undescribed by Degner. In one

* Edition of the Sydenham Society, 1844.

of his cases, he says, on the 4th day "the face indicated approaching death, algebat per totum corpus;" and in the 4th case (a recovery), in an old woman, he speaks of "intercurrent convulsions." But it is unlikely that he should have overlooked these symptoms had they existed in the majority of cases, and it is, moreover, unlikely that true cholera should have been prolonged to the 8th day. We do not feel much doubt that Dagner's epidemic was one of modified malarious dysentery, the relationship of which, both to common sporadic and epidemic malignant cholera, we have, however, already admitted.

2. *Dysentery with fevers of a typhoid character.* Cullen's definition of dysentery, which includes "pyrexia contagiosa," has been severely criticised by some more modern writers on dysentery. There is no doubt, however, that this definition is as correct when applied to certain well-marked epidemic attacks, as it is inapplicable to the common form of the disease. Cullen seems to have particularly referred to the epidemics recorded by Zimmermann and others; epidemics compounded of true dysentery with "a putrid fever." (Zimmermann, p. 10.)* In these and in similar cases, of which a very great number are on record, there is no doubt that dysentery was propagated from one person to another. The consideration of this point constitutes the chief and most important part of Dr. Harty's book, and we shall make no apology for considering it as fully as our limits will allow.

Dr. Harty starts with two propositions: 1st. That the combination above referred to really exists. 2d. That this combination alone is contagious. Our space will not allow us to give the evidence in detail, but the first proposition will, we think, meet with general assent.† The second is more doubtful, but we may anticipate our conclusions by saying, that in the great majority of cases in which there is clear evidence that the dysentery was contagious, there has been an accompanying low fever of some kind or other. Exceptions to this statement, which threw a doubt on its accuracy, are, however, brought forward by some late writers, particularly by Dr. Copland, who states, in his article on Dysentery, that under unfavorable circumstances among the dark races, "a putrid dysentery spreading rapidly through all breathing the impure air is developed, instead of an infectious typhus, which would be the result in the European constitution."

The belief that dysentery owes its contagious character to an accompanying fever is not only not a new one, but many of the best writers have taken great pains to enforce this point. We have already quoted Hoffman's opinion in the earlier part of our article. Etmuller‡ is no less explicit; he distinguishes dysentery into the benign and the malignant. "The benign form," he says, "is without fever, and also without contagion, and only sporadic; but the malignant dysentery is, for the most part, joined with a malignant and sometimes pestilential fever, and increases by contagion." This fever, he adds, is sometimes petechial. Zimmermann says, "Our dysentery was of itself not contagious" (p. 18.); but he after-

* The other chief authors to whom Cullen refers are Degner, Roederer, Grimm, Helwitch, Bontius, Huxham, and Cleghorn.

† Those of our readers who wish for more evidence on this preliminary position, and on the proofs of contagion, than our limits will allow, will find a short and well-chosen summary in the article Dysenterie, in the Dict. des Sciences Médicales, by Fournier and Vaidy.

‡ Opera Omnia, tom. ii, p. 149. Gen. 1697.

wards adds, "without possessing of itself any real malignity, still a dysentery becomes truly pestilential in foul and crowded hospitals, and of consequence much the more infectious." (p. 149.)* Bontius,† in his admirable definition of "True Dysentery," does not mention contagion, while in his account of the epidemic dysentery which arose when they were beseiged in Batavia by the people of Java, and which was attended by a petechial fever, he expressly mentions that it was contagious. It is needless to multiply quotations on this point; we shall merely add that several recent authors, particularly Vignes, have derived, from personal witness of the contagious form of dysentery, opinions almost identical.

The contagion of dysentery, therefore, is of that kind which has been lately termed "contingent," that is, not belonging to the disease itself, but impressed upon it by the presence of another disease, which at all times possesses the power of propagation. The fact that a disease may thus acquire a temporary power of propagation through the animal system appears undoubted; its explanation is a much more difficult point, and one which at present cannot be completely effected. It would be unwise in the present article to enter into a consideration of this subject, which would lead us at once into the whole question of contagion, considered with reference to continued fevers.‡

We pass on to a consideration of the nature of the fevers which, as far as can be determined from the accounts of writers, have accompanied the contagious form of dysentery. It would have pleased us better if Dr. Harty had used the term "contagious fever" instead of "typhus." The word typhus is employed in different senses by writers; some restrict it to a fever springing only from a specific contagion; others extend its application to any contagious fever, without regard to its origin. Dr. Harty seems to us to use it in both senses, and somewhat to obscure his meaning in consequence. Thus using the word in its restricted signification, he excludes certain epidemics from the list of contagious dysentery, because it is evident that in these cases the combined fever was of malarious origin. We allude more particularly to the epidemics recorded by Bontius and Clarke. (Harty, p. 109-18.) Again, when no doubt can be felt that a particular epidemic was really contagious, he asserts, unconditionally, that the attendant fever was typhus, and therefore, according to his notion, always arising from a single contagious virus; whereas, in certain instances, there is strong reason to believe that the fever, even when continued, was of malarious origin. As an example, we will select our author's examination of Roederer's account of the Morbus Mucosus, into which he enters at considerable length. We must, however, premise that we have no intention of entering into the previous question, as to

* In another place, however (pp. 133-4), Zimmermann, in reference to the distinction made between non-febrile and non-contagious, and febrile and contagious dysentery, remarks that "it has a dangerous tendency," but this opinion is evidently at variance with the whole bearing of the rest of his argument. On this point we are glad to find we agree entirely with Dr. Harty.

† Bontius, De Medicina Indorum, lib. iv, pp. 24-38. Paris, 1666.

‡ The words with which Roederer commences his work on the morbus mucosus are unfortunately too true, even at the present day: "Miræ sunt naturæ operationes, abdita atria, immensus rerum campus, limitata intellecta non emetiendus. Facile tamen persuademus omnia ad certas et perpetuas fieri leges, nihilque in tota rerum natura casu quodam fortuito et citra suas causes contingere. Sed quantillum in hoc amplissimo spatio ex hausit cognitio humana. Quot adhuc altissimis tenebris oblata, sibi soli intellecta natura nobis occultat. Quantus morborum numerus, quorum causæ, proxima æque et remota, abditæ in tanta rerum caligine procul dubio nos semper latebunt." (page 1.)

whether a contagious virus does arise during the prevalence of a malarious fever. We have already stated that the examination into this point is not our present purpose; we merely select for examination a particular epidemic which Dr. Harty admits to have been contagious.

The "morbus mucosus" which ravaged Gottingen in 1761, was a peculiar disease, allied, says Roederer, both to dysentery and to intermittent fever.* Various circumstances show, he says (p. 31), dysentery to be the offspring of intermittent, and as the dysenteric fever is the daughter of intermittent fever, so the subsequent epidemic morbus mucosus is an offshoot from this; and in subsequent pages he enlarges on the analogy both of dysentery and of the morbus mucosus to marsh fevers generally. Roederer asserts that both the dysentery and the morbus mucosus were highly contagious, and he does not limit this contagious property to one form of the disease but extends it to all. And it is evident that the morbus mucosus was allied to every grade of fever from a comparatively mild intermittent up to a most severe form, which he styles "febris mucosa, acuta, maligna, biliosa, putrida, soporosa." (p. 100.) A chapter is also devoted to its complication with scurvy. (p. 37.) Dr. Harty has taken so far the same view:

"The simple inference I draw from the rather complicated details of Roederer's work is this, that epidemic dysentery set in at the usual season; that this disease lapsed into the morbus mucosus, attended with every variety of fever, remittent, inflammatory, and malignant; that the dysentery affected chiefly the large intestines, while the other was an affection of the mucous follicles of the stomach and small intestines; and that both were highly contagious when in combination with typhus or malignant fever." (pp. 94-5.)

Now we cannot find that Roederer ever suspected that only one form, viz. that attended by the highest degree of malignant fever, was contagious. The concluding sentence is indeed Dr. Harty's own interpretation; it may be a correct one, that is not our point, it was not Roederer's opinion.

With regard more particularly to the dysentery, Roederer describes carefully two forms (pp. 5-8); the one slighter, with evening exacerbations; the other very severe, with bloody stools, dry fauces, red, rough, and dry tongue, or if moist, covered with a white and purulent mucus; frequent, small, feeble, impeded pulse; nausea, vomiting, and, at last, tympanitis; urine scanty, red or yellow, without cloud or sediment. In

* Roederer's description of the post-mortem appearances of this malarious and febrile dysentery agrees to some extent with Rokitansky's: "Intestina multum inflammata passim, gangrænosa, et quo ventriculo erant remotiora, eo majori in gradu depravata. Tunica villoso tenuium, veluti arte anatomica injecta, vasculis pictis distincta, copiosissimis punctis parvisque areolis nigris conspersa. Superficies interna crassorum lacera, inequalis, *igne quasi combusta*, obscure rubra quia nigricans."

We shall also insert his account of the large intestine in the morbus mucosus, as given in the dissections at the end of the volume (p. 255). In the 1st case, the intestine was gangrenous; in the 2d, not inflamed, but contracted; in the 3d, the intestine being macerated in water for some days and the blood washed off, the lining membrane was found rough, with many little elevations or papillæ, irregular or wartlike, quasi verrucosis." In the 4th case, slightly inflamed "inflammata;" in the 5th case, numerous "copiosissimi folliculi coagmentati" were found in the ileum, cæcum, and ascending colon. They had black orifices, and were both aggregated and distinct. In the 6th dissection, the mucous follicles were copious, large, distended (repleti), surrounded by a red circle. In the 7th and 9th dissection he mentions merely the abundance of the worms (Trichinodes); the same also in the 8th case, but also "in the cæcum and appendix vermiformis the follicles were seen with black points." In the 10th, the tunica was without notable inflammation. In the 11th case, the ascending colon was gangrenous. In the 12th, "inflamed." In this morbus mucosus the small intestines were much more diseased than the large. There was a great formation of mucus and an abundance of small worms, resembling ascarides; lumbrici were also common.

the worst forms there were black gangrenous maculæ, and other signs of a most malignant fever. In speaking of contagion, no distinction is made between these two forms.

Another epidemic of decidedly malarious origin was that described by Morton, and to which we have already alluded. That Morton believed this to be contagious is certain, for he tells us, that in the month of August he was himself attacked after incautiously inspecting the stools of a dysenteric patient, and in the following autumn he was again attacked from the same cause.

With regard to the "putrid dysentery," witnessed by Clarke,* in Bengal, and which was evidently a combination of dysentery with the common Pucca or endemic remittent fever, Dr. Harty decides that Clarke's positive assertion, that the disease was contagious, must have been erroneous. The chief reason which induced him to form this opinion is, that Clarke held heterodox notions about fevers generally, believing even intermittents to be *slightly contagious,* and asserting that malignant remittents became continued. Therefore Dr. Harty concludes "that the person who entertained such opinions could have no hesitation in pronouncing the remittent form of dysentery to be always contagious. Having thus disposed of his testimony, we proceed to others of a less equivocal character." (p. 120.) We are afraid that if Clarke had written "typhus" instead of "remittent" fever, his testimony would have been dealt with more mercifully. Other epidemics, however, have been manifestly attended with a fever unequivocally belonging to the "typhus family." We need only mention those described by Sennertus, Zimmermann, Grimm, Rouppe, and, in our own times, Vignes and Dewar. We conclude, therefore, that contagious dysentery, prevailing epidemically, has been accompanied always by some kind of contagious fever. But if we are to receive the accounts of highly respectable writers as sufficient evidence, there is reason to believe that this contagious fever was occasionally of malarious origin. In several places, indeed, Dr. Harty appears to adopt this view; thus he says, in reference to Morton's epidemic: "We may fairly conclude that its contagious property arose under similar circumstances, that is, by the frequent conversions of bad remittents into continued fevers of a contagious type, a species of conversion, as we have just seen, strongly insisted on by Clarke." (p. 121.)† And yet in the previous page he has strongly condemned Clarke for holding this very opinion. In several other places Dr. Harty admits unconditionally that a fever arising from malaria, and consequently of remittent type, in the first instance, may become contagious. "That fever may, as we have repeatedly proved, be either originally a malignant typhus, or it may have been a *remittent* of bad character, converted into one of a continued contagious type. Such a change, though doubted by Bancroft, is too well authenticated to be called seriously into question." (p. 154.) This is quite in accordance with our views, but we object to the term typhus being applied to a fever of malarious origin, and which does not present the anatomical signs of true typhus.

It is worthy of notice, that when this contagious fever has apparently arisen from malaria, there have been present symptoms of scurvy in many cases, showing the general prevalence of causes exerting a manifest de-

* Observations on the Diseases which prevail in Long Voyages to Hot Countries, p. 318. 2d edition. London, 1792.

† Diseases of Hot Climates, 1809

teriorating effect on the nutritive processes. And sometimes symptoms have occurred which seem to show that there were tendencies towards other blood-diseases, for example, sweating sickness and a species of cholera. Several instances of this occur in the works of Roederer, Grimm, and others.

Dr. Copland's opinions are somewhat different from those we have now stated :*

"Many writers," he says, "conceive that the asthenic varieties described above are complications of simple dysentery with different kinds of fever, and that when they are infectious it is not the dysentery, but the fever which possesses this property. Some authors suppose that the typhoid variety especially is a complication of this description ; but if such be the case, wherefore should the disorder which is communicated be always dysentery and not fever ? Moreover, this form of dysentery is often present where a case of typhus cannot be found." (p. 705.)

With regard to the first statement, that the disorder communicated is always dysentery and not fever, Dr. Harty opposes the observation of Zimmermann, who remarks : "When hospitals are filled with dysenteric people, some of the assistants are attacked only with the dysentery, and others with the gaol or hospital fever, that ends in bloody and gangrenous stools." (p. 151.)

Dr. Copland's second argument, that the low or so-called typhoid dysentery is often present without any accompanying typhus, is thus answered by Dr. Harty :

"For this statement no authority is given, such as might be investigated ; but is not Dr. Copland well aware that, under favorable circumstances (such as those recapitulated by Frank and others), there is no disease more readily superinduced or spontaneously generated than typhus, a position fully supported by the several authorities already quoted in illustration of this very typhoid variety." (p. 139.)

We have so much respect for any statement made by Dr. Copland respecting dysentery, that we have thus placed his opinion in opposition to the almost unanimous evidence on the other side of the question. We believe that this opinion arises in part, at least, from Dr. Copland's view of the contagion of typhus, which he does not allow to be generated so easily by the concurrence of certain circumstances as Dr. Harty supposes. But we confess that we feel little doubt that on this point Dr. Copland is mistaken, and that Dr. Harty's position, with perhaps the modification we have ventured to introduce, is the correct one.

The question whether the asthenic varieties of dysentery are produced by the concurrent action of a febrile disease, is answered in the negative by Dr. Copland :

"The fact is incontrovertible, that the asthenic forms, some of which are considered as complications by many writers, are direct and necessary and uniform results of certain diversified but concurrent causes, and not contingent associations of two diseases capable of separate existences." (p. 705.)

These concurrent causes are stated to be "cold and moisture, malaria, unwholesome food and water, or emanations contaminating the fluids."

We are convinced that this is the true mode of viewing the asthenic dysentery, viz. in reference to the causes which produce it. "It sufficently appears," says Van Swieten, "that the dysentery is multiplex as the causes whence it proceeds" (tom. iii, p. 193) ; and he proceeds to add, that

* Dictionary of Practical Medicine, art. Dysentery.

when the powers of life are prostrated in the commencement of the disease, this is not from exhaustion on account of the quantity of excreted matter, but from some poisonous miasma. Admitting that these causes do really produce a low form of dysentery, without concomitant fever, we can readily believe that occasionally, and indeed, often, fevers are present, because these causes are also active in their production. Sydenham's expression may with equal justice be applied here, and the asthenic dysentery be called a "febris introversa."

Doubts still exist whether the contagion of dysentery is derived from the stools only, or from exhalations given off indifferently from the lungs, skin, &c., as is presumed to occur in typhus or the contagious exanthemata. Dr. Harty, of course, adopts this last view, although he does not deny that the contagious virus may be also developed from the excretions. These certainly seem in some cases to be the "foyers" of the contagious effluvia. Thus Pringle relates, in the epidemic dysentery which prevailed after the battle of Dettingen, and which arose apparently from sudden changes of temperature and great exposure of the troops, and afterwards became contagious from development of typhus or hospital fever, that when they left the village which had been converted into an hospital, and which was filled with the dirty straw covered with the dysenteric excretions, the disease ceased immediately to be propagated by contagion. Most of the older writers believed the effluvia from the evacuations to be contagious. Fabricius Hildanus, in his work, 'De Dysenterica,' states this very strongly; so also Sennertus, who believed also that the breath of a dysenteric patient could communicate the disease. Vignes remarks: "We have seen many sick contract the disease in going to the common places where the dysenterics render their stools." (p. 205.)

Chomel holds the same opinion. (Diction. de Médecine, tom. x, p. 129.)

It must be remembered that it is only in the dysentery with malignant fever that the stools have been declared capable of communicating the disease. In common sporadic cases this never occurs, as is proved by daily observation. It is true that Lind, in his first paper on "Infection," tells an extraordinary story of a patient with chronic flux infecting almost all who used the same necessary with him, but this is a very doubtful case, and some few others, of a similar nature, which are recorded, are equally apocryphal.*

3. *Dysentery with other abdominal diseases.* We have entered at such length into the previous complications with fevers, that we must shorten our remarks on the combined abdominal diseases. A connexion between dysentery and certain diseases of the liver has been long taught by writers, although the exact steps of the connexion, and even the relative bearing to each other of the two diseases, are still matters of doubt. Dr. Harty, rather to our surprise, passes the whole subject without notice. Dr. Morehead notices as complications: 1st, with peritonitis; 2d, with morbid lesions of the small intestines, which, we are told, "are not very common, but when it does occur, is generally found at the termination of the ileum,

* At p. 260 Dr. Harty quotes Hildæus Podoanus: "Eo malo, inquit, sæpius videas corrupi, quibus clyster infunditur, instrumento non bene abluto quo antea dysentericus usus fuit; in sedili etiam seu loco excretionis contagii vestigia aliquando remanent." "The analogy of hospital gangrene," observes Dr. Harty, "in which both a general and local infectious power exists, would make me hesitate in refusing to admit a similar power in dysentery." (p. 260.)

and consists in ulceration of Peyer's glands; there is also sometimes a granular lymphy secretion." (Op. cit. p. 146.) 3d. With abscess in the liver, which is most common in the form of the disease leading to circular follicular ulcers. (p. 143.) On this subject Dr. Morehead remarks:

"Complication with abscess in the liver constitutes a great proportion of the fatal cases of chronic dysentery. In one set of cases, those ranged by me under the head of dysentery, the affection of the bowels was the primary and prominent disease; that of the liver coming on more or less obscurely as a secondary event. In the other set, the symptoms of hepatitis were the primary and most prominent, those of ulceration ef the bowels succeeding, and being not very clearly indicated." (Op. cit. p. 149.)*

Of 30 fatal cases of dysentery, recorded in the same number of the Transactions by Dr. Morehead, we find twelve to have been attended by hepatic abscess. In contrast with this statement, we may put the following remark of Dr. Baly:

"Among the many hundreds of cases of dysentery which have occurred in the Milbank Prison, during the last seven years, not one has been complicated with hepatic abscess. The medical records of the establishment too, which reach back to the year 1824, affords no grounds for even a suspicion that such cases ever occurred among the prisoners." (p. 16.)

Dr. Baly explains this discrepancy of statement by supposing that "the malaria causing the dysentery, has at sometimes, and in some places, the property of predisposing to abscess of the liver, and at other times, and in other places has not this property." (Op. cit. p. 17.) He also attributes considerable influence to the effect of simple and spare diet in counteracting the tendencies towards hepatic disease. Into a full consideration of this important question, it is impossible now to enter. A superficial inquiry would be worse than useless, but we believe, that did our space allow us, we could explain some of the difficulties of the inquiry.† We will at this time merely remark, that too much attention seems to have been given to abscess merely of the liver, without reference to other diseased conditions of the organ, such as simple congestion, enlargement, granulation, &c. Those who contend that secondary hepatic abscess is owing to absorbtion of pus, or of some hurtful matter from an ulcerated mucous membrane, or to a true phlebitis, seem to us too narrow in their views of dysentery and liver diseases. They have confined themselves to one phenomenon, and have disregarded other necessary conditions of the question.

That abscess of the liver occasionally, and indeed often arises from the causes now specified, viz. phlebitis and purulent absorbtion, is an undoubted

* With reference to the cicatrization of colonic ulcers with hepatic abscess, Dr. Morehead says: "It is a satisfactory fact, that cicatrization of ulcers of the mucous coat of the intestine will go on under circumstances which *a priori* would be considered most adverse, e. g. under the coexistence of abscess of the liver." This agrees with the observations of Parkes and others.

† The experience of dysentery of the hospital ship "Minden," during the Chinese war, as detailed by Dr. Wilson, has been considered decisive as to the non-existence of hepatic abscess with that form of dysentery. But on this point it is necessary to wait for more evidence. It is premature to consider the peculiar and multiform diseases occurring during a campaign as proper cases for inquiries of this kind. Sir James M'Grigor has, with his accustomed liberality, permitted us to look over the admirable Reports sent home from China. It is evident from these, that during the campaign, the troops suffered from the several well-marked forms of malarious dysentery, either alone or combined with scurvy. Since they have been in barracks and cantonments, the Chinese dysentery is stated by several gentlemen to be assuming gradually the forms of common Indian dysentery, and to be complicated with abscess and other diseases of the liver. But the subject is too important to be discussed here.

fact—that they will account for all cases of abscess we cannot admit. Even Cruveilhier, who may be considered as one of the originators of this doctrine, never carried it to such an exclusive pitch as some of his followers have done. (See Livraison xl, p. 5.)

We cannot close our examination of this subject without noticing some extraordinary nervous symptoms observed by Dr. Baly at Milbank. Similar symptoms prevailed in the Penitentiary in 1823, and are detailed by Dr. Latham. These were at that period chiefly, "headache, vertigo, cramps, and twitchings of the limbs, delirium, convulsions, and apoplexy." Dr. Baly has noticed cramps, chiefly of the extremities, and diminution of sensation in several cases; in other instances, there has been stupor and pseudo-hysterical symptoms. In one case there was a kind of modified cataleptic ecstacy. A man, aged 22, was attacked with profuse serous diarrhœa, possibly that modification of malarious dysentery to which we have already alluded. Bleeding, calomel, and opium proving inefficacious, metallic astringents, (sulphates of copper and zinc, we presume,) with opium, were employed.

"The new remedies immediately arrested the diarrhœa, but now a fresh train of symptoms presented themselves. The patient's mind seemed no longer to take cognizance of the impressions made on his senses. He sat up in bed frequently repeating half aloud a sentence consisting of two or three words. His eyes were open, but he did not seem to observe surrounding objects; when questions were put to him, he did not answer, or only repeated the last word or two of the question. When his chin was depressed, he mechanically protruded his tongue and kept it protruded until his lower jaw was raised again; he then withdrew his tongue and allowed his mouth to be closed. When his arm was raised, he kept it in the position given to it, till it was returned to its former place by another person. Food and medicine he swallowed when they were put into his mouth, but never expressed repugnance for the one or desire for the other. He appeared conscious of no suffering; his skin was cool, his tongue moist, pale, and nearly clean, and his pulse slow and rather full. In this state he continued three days; the nervous symptoms then passed off, and merely slight diarrhœa remained." (pp. 22-3.)

Dr. Baly remarks, that these nervous symptoms were merely "functional," that is, unattended by appreciable organic changes after death; and he ascribes their prevalence at Milbank to the general increased excitability of the nervous system, induced by long confinement. Prisoners, who have been long in prison, and are then suddenly transferred to the convict ships in the river, and thrown at once from perfect order and repose, into scenes of excitement, are occasionally subject to violent epileptiform convulsions. The same excitability which renders them liable to these attacks, may render them more liable to excito-motory actions when attacked by diarrhœa or dysentery in prison.

But we must bring our remarks to a conclusion, and regret that our limited space allows us to go into no detail on the many interesting subjects we have been compelled so cursorily to review. The symptoms and treatment of dysentery, detailed by Drs. Baly and Morehead, although highly important, present nothing unusual, or that call for special notice. Dr. Harty devotes 100 pages to the treatment of dysentery, and gives us a valuable account of the method adopted by many of the best writers. But those who have time to refer to this subject will do well to consult the work itself.

